# The relationship of dietary total antioxidant capacity with sarcopenia and cardiometabolic biomarkers in type 2 diabetes patients

**DOI:** 10.14814/phy2.15190

**Published:** 2022-02-12

**Authors:** Nadya Baharirad, Yahya Pasdar, Mostafa Nachvak, Saeid Ghavamzadeh, Ali Soroush, Amir Saber, Shayan Mostafai, Armin Naghipour, Hadi Abdollahzad

**Affiliations:** ^1^ Student Research Committee School of Nutritional Sciences and Food Technology Kermanshah University of Medical Sciences Kermanshah Iran; ^2^ Department of Nutritional Sciences School of Nutritional Sciences and Food Technology Kermanshah University of Medical Sciences Kermanshah Iran; ^3^ Department of Nutrition, Medicine Faculty Urmia University of Medical Sciences Urmia Iran; ^4^ Cardiovascular Research Center Health Institute Kermanshah University of Medical Sciences Kermanshah Iran; ^5^ Research Center for Environmental Determinants of Health (RCEDH) Kermanshah University of Medical Sciences Kermanshah Iran; ^6^ Clinical Research Center Imam Reza Hospital Kermanshah University of Medical Sciences Kermanshah Iran

**Keywords:** antioxidants, blood biomarkers, DTAC, muscle strength, sarcopenia, type 2 diabetes

## Abstract

**Background:**

The aim of this study was to investigate the relationship of dietary total antioxidant capacity (DTAC) with sarcopenia and metabolic biomarkers in people with type 2 diabetes in the Kurdish race.

**Methods:**

In this cross‐sectional study, data of 189 type 2 diabetic patients (35–65 years old) from RaNCD cohort study were evaluated. DTAC, fasting blood sugar, lipid profile, body composition, muscle strength, and sarcopenia were assessed. *t* and χ^2^ tests to compare the variables between sarcopenic and non‐sarcopenic patients and one‐way analysis of variance to compare the variables in DTAC tertiles were used. The relationship between DTAC and different variables was evaluated using multiple logistic regression model.

**Results:**

The mean age and body mass index were 49.7 ± 8.7 years and 27.1 ± 3.9 kg/m^2^. Body mass index, waist circumference, and hip circumference were significantly different between diabetic patients with and without sarcopenia (*p *< 0.05). In crude (*p *= 0.010) and adjusted (*p *= 0.035) models, there was a significant relationship between DTAC and fasting blood sugar. Also, the relationship between DTAC with waist (*p *= 0.019) and hip (β = −4.25, *p *= 0.026) circumference was significant. Sarcopenia was significantly lower in the third tertile in comparison with the first tertile of DTAC (*p *= 0.016).

**Conclusion:**

Diet with higher DTAC can be associated with lower fasting blood sugar, abdominal obesity and sarcopenia in type 2 diabetic patients. However, further studies are required to confirm these relationships.

## Graphical abstract

Higher DTAC is associated with better FBS in type 2 diabetic patients. Anti‐inflammatory diet is associated with less central obesity and sarcopenia in patients with type 2 diabetes.

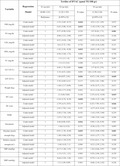

## 1 INTRODUCTION

Sarcopenia is an involuntary resorption of skeletal muscle with decreased muscle strength and function (Sun et al., 2021), which has recently been considered as a new complication in diabetic patients (Cruz‐Jentoft et al., 2019; Sluijs et al., 2015; Yasemin et al., 2019). The pathogenesis of sarcopenia is complicated and associated with aging, sedentary lifestyle, nutritional factors, increased production of reactive oxygen species, increased levels of proinflammatory cytokines, hormonal changes, and decreased neuromuscular function (Chen et al., 2020; Tan et al., 2021; Trierweiler et al., 2018). Age‐related causes of sarcopenia are related with chronic inflammation due to oxidative stress and proinflammatory cytokines (Lima et al., 2019). Moreover, there are some nutritional causes for incidence of sarcopenia include inadequate energy and protein intake, low blood levels of vitamin D, and inadequate intake of carotenoids and antioxidants (Yakout et al., 2019). Besides, sarcopenia is associated with an increase in clinical side effects such as the risk of physical disability, decreased quality of life, poor response to various treatments, and eventually premature death. Some studies have shown that the rate of sarcopenia and subsequent physical disability is higher in patients with diabetes type 2 (Galarregui et al., 2018; van der Schaft et al., 2019). The most important mechanisms that are responsible for sarcopenia in diabetic patients include impaired protein synthesis (Cuthbertson et al., 2017), insulin resistance, and oxidative stress (Sugimoto et al., 2019; Trierweiler et al., 2018). Furthermore, the findings of several investigations that assessed the relationship between dietary total antioxidant capacity (DTAC) with lipid profile disorders, hypertension, sarcopenia, as the complications of type 2 diabetes, were inconsistent (Abbasi et al., 2019; Cuthbertson et al., 2017; Gunawardena et al., 2019; Sluijs et al., 2015; Sugimoto et al., 2019). Some studies suggested that antioxidants such as carotenoids and vitamins (α‐, β‐ and γ‐tocopherol and vitamin C) can be effective in reducing risk of type 2 diabetes via decreasing oxidative stress (Abbasi et al., 2019; Ceriello & Motz, 2004; Johansen et al., 2005; Psaltopoulou et al., 2010). However, these findings are not supported by randomized, controlled trials of vitamin supplements (Group HPSC, 2002; Montonen et al., 2004). As well, Abbasi et al. evaluated the relationship between DTAC and kidney diseases in subjects with type 2 diabetic. Although there is compelling evidence for the role of oxidative stress in development of kidney diseases in diabetic patients, DTAC showed no significant association in these patients (Abbasi et al., 2019; Earle et al., 2016). Several observational, clinical trial, and cohort studies have demonstrated that β‐carotene and lycopene, as natural dietary antioxidants, have inverse effects on diabetes, and their supplementation has no effects on diabetes progression (Ärnlöv et al., 2009; Kataja‐Tuomola et al., 2011; Montonen et al., 2004; Song et al., 2009). Also, Sugimoto et al. indicated that there is a linear relationship between hyperglycemia and poor glycemic control with the frequency of sarcopenia and low muscle mass in type 2 diabetic patients (Sugimoto et al., 2019). Regarding these inconsistent findings, the present study was carried out to determine the relationship between DTAC with sarcopenia and blood biomarkers in people with type 2 diabetes in the Kurdish race, in Iran.

## 2 MATERIALS AND METHODS

### 2.1 Study design and data collection

The present study is a cross‐sectional descriptive‐analytical study that used the data of the initial phase of the prospective study of non‐communicable diseases of Ravansar (RaNCD), which is a part of prospective epidemiological research in Iran (PERSIAN). The RaNCD study is a population‐based prospective study that has been conducted on the 10,065 men and women aged 35–65 years to investigate the results of chronic diseases and death in Ravansar city since 2014 (Eghtesad et al., 2017; Poustchi et al., 2018). All of the ethnically Kurdish patients with type 2 diabetes that participated in RaNCD were included in the study. Also, people with type 1 diabetes, any type of cancer, heart attack or stroke, kidney failure, pregnant or lactating women, people taking hormonal or steroid medications, people with incomplete or lost information were excluded from the study. Finally, by considering the inclusion and exclusion criteria, 189 patients met the eligibility criteria and after filling the written consent form were included in the study. All demographic and clinical information of the participants was recorded, and they were assured that their information would be kept confidential. In the next step, all of the participants were referred to the laboratory of the Cohort Center and blood samples were taken. Samples were collected for biochemical tests including Fasting blood sugar, Total cholesterol (TC), Triglyceride (TG), Low density lipoprotein (LDL), and High‐density lipoprotein (HDL). Height was measured without shoes by a standard stadiometer (BSM370) with an accuracy of 0.1 cm and weight was measured with light clothes using Inbody 770 body composition analyzer. Waist and hip circumference were measured using an inelastic tape measure without applying any pressure to the body; then waist to hip ratio was calculated. Body composition including percentage of body fat, body fat mass, fat free mass, skeletal lean mass, skeletal muscle mass (SMM), and total body water were measured by body analyzer (Inbody 770). Systolic and diastolic blood pressure were measured using standard manual sphygmomanometer (Riester), after at least 4–5 mins of rest in sitting position, two times from both arms and the interval between two measurements was 10 mins. The mean of the two obtained blood pressure from both arms were recorded as the final blood pressure. To evaluation of the physical activity status, the physical activity questionnaire designed for PERSIAN Cohort was used to assess the participants’ physical activity. This questionnaire included 21 questions about the number of different activities of the person during the day and night, the answers to these questions were recorded in hours or minutes per day in the questionnaire. One of the advantages of this questionnaire is the possibility of calculating a numerical or quantitative measures for each physical activity called the MET (Metabolic equivalent of task) index (Frehlich et al., 2019).

### 2.2 Sarcopenia diagnosis

The diagnosis of sarcopenia was based on the measurement of appendicular muscle mass (AMM) by bioelectrical impedance analyzer and the measurement of muscle strength using a handgrip dynamometer. AMM is correlated with body size and can be adjusted for body size in different ways, i.e. using AMM/height^2^ as skeletal muscle index (SMI) (Cruz‐Jentoft et al., 2019; Moon et al., 2018). SMI values less than 7.26 kg/m² for men and 5.45 kg/m² for women are considered as decreased muscle mass or pre‐sarcopenia (Trierweiler et al., 2018). Sarcopenia was defined as decreased SMI and muscle strength (values less than 27 kg for men and 16 kg for women) (Bianchi et al., 2016).

### 2.3 Absolute and relative muscle strength

The muscle strength of the hand was measured with a standard hand dynamometer from the dominant hand. Measurements were taken twice in a sitting position with the elbow angle at a vertical axis of 90° (Li et al., 2018). Relative muscle strength was calculated by dividing the maximum absolute muscle strength by body mass index (BMI) (Lawman et al., 2016).

### 2.4 Dietary total antioxidant capacity

Dietary intakes were assessed using a food frequency questionnaire by face‐to‐face interview. According to the reference table that indicates the amount of food antioxidant content in terms of 100 g of foods (Haytowitz & Bhagwat, 2010), the daily amounts of food antioxidants were calculated (Mirmiran et al., 2017). The antioxidant content of foods was determined according to oxygen radical absorbance capacity (ORAC) method that has been already approved by the U.S. department of agriculture (USDA) (Harasym & Oledzki, 2014). This method expresses the degree and duration of inhibition of oxidation induced by proxy radicals and is equivalent to Trolox (Haytowitz & Bhagwat, 2010). To determine the DTAC of local foods, a meeting was held with the presence of expert nutritionists and according to USDA report the amount related to each food item was estimated.

### 2.5 Data analysis

In this study, quantitative and qualitative variables are reported as mean ± standard deviation (SD), and number (percentage), respectively. The obtained DTAC values were categorized into tertiles. The normality of the data was assessed using the Kolmogorov‐Smirnov test. The comparative tests for quantitative and qualitative variables were t‐test and chi‐square test (χ^2^), respectively. The differences between variables among DTAC tertiles were analyzed using a variance test.

The logistic regression model was performed to investigate the relationship between DTAC and variables. The effect of confounding variables such as age, sex, BMI, physical activity, and total calorie intake were adjusted in the model. Statistical analysis was performed using SPSS software version 16. For all tests, *p*‐values less than 0.05 were considered statistically significant.

## 3 RESULTS

In the present study, after considering the exclusion criteria, 189 type 2 diabetic patients including 80 men (42.3%) and 109 women (57.7%) with a mean age of 49.7 ± 8.7 were included. The BMI amounts of the patients were 27.1 ± 3.9 kg/m^2^, that was significantly different between type 2 diabetic patients with and without sarcopenia (*p *< 0.05). Also, a significant difference was found among the variables of educational level, physical activity level, height, BMI, waist and hip circumference values between groups (sarcopenic and non‐sarcopenic) (*p* < 0.05) (Table 1).

**TABLE 1:** Comparison of mean quantitative variables and frequency (percentage) of qualitative variables in sarcopenic and non‐sarcopenic type 2 diabetic patients

Variable		Total (*n* = 189) (100%)	Sarcopenia, *n* (%)		*p*‐value
			Yes	No	
			108 (57.1%)	81 (42.9%)	
Level of education	Illiterate	59 (31.2)	22 (20.4)	37 (62.7)	0.001
	Elementary	99 (52.4)	58 (53.7)	41 (50.6)	
	High school	16 (8.5)	15 (13.9)	1 (1.2)	
	University	15 (7.9)	13 (12.0)	2 (2.5)	
Residence type	Urban	4 (2.1)	2 (1.9)	2 (2.5)	0.77
	Rural	185 (97.9)	106 (98.1)	79 (97.5)	
Physical activity	Low	64 (33.9)	36 (33.3)	28 (34.6)	0.035
	Moderate	61 (32.3)	28 (25.9)	33 (40.7)	
	Sever	64 (33.9)	44 (40.8)	20 (24.7)	
Absolute muscle strength	Normal	120 (63.50)	67 (62.0)	53 (65.4)	0.373
	Weak	69 (36.5)	41 (38.0)	28 (34.6)	
Relative muscle strength		1.3 ± 0.5	1.2 ± 0.4	1.4 ± 0.5	0.918
Age		49.72 ± 8.7	49.6 ± 8.6	49.7 ± 8.9	0.081
Weight (Kg)		71.12 ± 3.3	73.12 ± 3.6	68.11 ± 8.7	0.067
Height (Cm)		162.9 ± 0.3	167.7 ± 5.4	154.5 ± 6.9	0.001
BMI (Kg/m^2^)		27.12 ± 3.89	26.1 ± 3.5	28.6 ± 4.3	0.003
WC (cm)		103.7 ± 1.4	102.6 ± 0.7	104.8 ± 6.0	0.019
HC (cm)		98.9 ± 9.3	97.9 ± 7.2	100.9 ± 7.2	0.026
WHR		1.1 ± 0.1	1.1 ± 0.1	1.1 ± 0.1	0.378
Total calorie intake		3261.11 ± 184	3358.2.3 ± 108	3146.91 ± 301	0.226
SBP (mmHg)		111.17 ± 1.4	111.8 ± 1.2	110.9 ± 16.2	0.981
DBP (mmHg)		71.1 ± 9.8	71.1 ± 10.4	70.9 ± 8.9	0.886

Note

Values are expressed as mean ± standard deviation and number (percent).The p values <0.05 was considered as significant with independent T test and chi‐square test (χ2).

Abbreviations: BMI, body mass index; WC, waist circumference; HC, Hip circumference; WHR, waist to hip ratio; SBP, systolic blood pressure; DBP, diastolic blood pressure.

Moreover, the prevalence of sarcopenia and weak absolute muscle strength was 57.1% (*n* = 108), and 36.5% (*n* = 69) in diabetic patients. The mean values of fasting blood sugar, TG, LDL, HDL, and TC were 139.8 ± 34.3, 161.4 ± 74.9, 106.2 ± 4.3, 43.1 ± 4.6, and 182.9 ± 37.2, mg/dL respectively, which did not show a statistically significant difference among the DTAC tertiles (*p *> 0.05) (Table 2). Besides, there were no significant differences between ALT, AST, weight, height, BMI, waist circumference, hip circumference, waist to hip ratio, SBP, DBP, having sarcopenia, and absolute muscle strength between tertiles in type 2 diabetic patients.

**TABLE 2:** Comparison of mean blood biomarkers, anthropometric indices, blood pressure, sarcopenia, absolute and relative muscle strength based on DTAC tertiles in type 2 diabetes

Variable		Mean ± SD *n* = 189	Tertiles of DTAC (μmol TE/100 gr)			*p*‐value
			T1 (*n* = 63)	T2 (*n* = 62)	T3 (*n* = 64)	
			0.88–1.21	1.22–155	1.56–2.24	
FBS (mg/dl)		139.8 ± 34.3	146.4 ± 64.3	134.9 ± 24.2	138.1 ± 27.9	0.158
TG (mg/dl)		161.4 ± 74.9	154.7 ± 63.5	156.2 ± 79.6	173.4 ± 93.7	0.358
LDL (mg/dl)		106.2 ± 4.3	107.3 ± 6.2	105.2 ± 9.1	105.2 ± 6.1	0.911
HDL (mg/dl)		43.1 ± 4.6	45.1 ± 2.3	42.8 ± 0.5	43.1 ± 0.7	0.233
TC (mg/dl)		182.9 ± 37.2	185.3 ± 21.5	182.4 ± 1.2	181.3 ± 4.1	0.068
ALT (IU/L)		19.4 ± 5.9	19.1 ± 6.5	19.3 ± 5.8	20.3 ± 5.9	0.767
AST (IU/L)		22.9 ± 9.9	21.1 ± 9.4	22.1 ± 3.0	21.6 ± 1.2	0.891
Weight (Kg)		71.1 ± 3.3	73.1 ± 8.0	68.1 ± 3.0	71.1 ± 9.4	0.172
Height (Cm)		161.9 ± 9.3	160.8 ± 4.0	161.1 ± 8.4	163.9 ± 6.2	0.174
BMI (Kg/m2)		27.3 ± 1.9	28.3 ± 4.6	26.4 ± 1.7	26.3 ± 6.1	0.079
WC (cm)		103.7 ± 1.6	104.7 ± 5.6	102.7 ± 8.1	102.7 ± 0.1	0.156
HC (cm)		98.9 ± 9.4	100.9 ± 3.1	99.8 ± 4.9	97.9 ± 2.9	0.161
WHR		1.1 ± 0.1	1.1 ± 0.1	1.1 ± 0.1	1.1 ± 0.1	0.509
SBP (mmHg)		111.0 ± 19.2	110.9 ± 17.0	109.4 ± 16.2	112.8 ± 19.2	0.567
DBP (mmHg)		71.0 ± 9.9	71.4 ± 9.5	70.3 ± 9.7	71.5 ± 10.5	0.755
Sarcopenia	Yes	106 (57.0)	30 (48.4)	34 (54.0)	42 (68.9)	0.061
	No	80 (43.0)	32 (51.6)	29 (46.0)	19 (31.1)	
Absolute muscle strength (Kg)	Normal	119 (63.6)	44 (71.0)	38 (59.4)	37 (60.7)	0.337
	Weak	68 (36.4)	18 (29.0)	26 (40.6)	24 (39.3)	

Note

Values are expressed as mean ± standard deviation and number (percent).

The *p* values <0.05 was considered as significant with one‐way analysis of variance and chi‐square test (χ^2^).

Abbreviations: BMI, body mass index; DBP, diastolic blood pressure; DTAC, dietary total antioxidant capacity; FBS, fasting blood sugar; HC, Hip circumference; HDL, high density lipoprotein; LDL, low density lipoprotein; SBP, systolic blood pressure; TC, total cholesterol; TG, triglycerides; WC, waist circumference; WHR, waist to hip ratio.

As shown in Table 3, in the logistic regression analysis, the crude and adjusted models indicated that fasting blood sugar in the second tertile of DTAC was 1.53 and 2.63 time lower than the first tertile (*p *= 0.010, *p *= 0.035). In the crude model, TG in the third tertile was 1.07 higher than the first tertile (*p *= 0.001), however after adjustment for confounding factors the ratio was 1.53 time lower (*p *= 0.166). Although the values of LDL, HDL, and TC in the second and third tertiles in both models were lower compared to the first tertile, only the ratio of HDL in the crude model of the second tertile was significant (*p *= 0.015).

**TABLE 3:** Association of DTAC with blood biomarkers, anthropometric indices, sarcopenia, absolute and relative muscle strength in patients with type 2 diabetes

Variable	Regression Model	Tertiles of DTAC (μmol TE/100 gr)				
		T1 (*n* = 63)	T2 (*n* = 62)	*p*‐value	T3 (*n* = 64)	*p*‐value
		(0.88–1.21)	(1.22–1.55)		(1.56–2.24)	
		Reference	β (95% CI)		β (95% CI)	
FBS (mg/dl)	Crude model	1	−1.53 (−2.69, −0.37)	0.010	−0.9 (−1.23, 1.05)	0.875
	Adjusted model	1	−2.63 (−5.08, −0.19)	0.035	−1.18 (−3.59, 1.24)	0.340
TG (mg/dl)	Crude model	1	0.37 (−0.98, 0.24)	0.236	1.07 (0.44, 1.71)	0.001
	Adjusted model	1	−0.06 (−2.21, 2.09)	0.957	−1.53 (−3.69, 0.63)	0.166
LDL (mg/dl)	Crude model	1	−0.51 (−0.56, 1.58)	0.352	−0.93 (−2.01, 0.16)	0.093
	Adjusted model	1	−0.4 (−2.72, 1.92)	0.736	−1.85 (−4.18, 0.48)	0.121
HDL (mg/dl)	Crude model	1	−1.42 (−2.56, −0.28)	0.015	−0.03 (−1.09, 1.15)	0.963
	Adjusted model	1	−0.56 (−2.84, 1.72)	0.086	−2.01 (−4.31, 0.29)	0.730
TC (mg/dl)	Crude model	1	−3.3 (−2.5, 1.8)	0.080	−4.3 (−2.4, 7.7)	0.081
	Adjusted model	1	−1.5 (−2.5, 0.5)	0.208	−1.6 (−2.7, 2.5)	0.173
ALT (IU/L)	Crude model	1	0.44 (−1.78, 0.89)	0.026	1.57 (0.19, 2.95)	0.519
	Adjusted model	1	3.15 (−0.51, 6.82)	0.092	1.01 (−2.59, 4.61)	0.583
AST (IU/L)	Crude model	1	1.04 (0.07, 2.01)	0.036	−0.97 (−1.94, −0.01)	0.049
	Adjusted model	1	2.76 (−0.69, 6.21)	0.117	0.59 (−2.81, 3.98)	0.735
Weight (Kg)	Crude model	1	−1.04 (−2.17, 0.09)	0.498	−0.39 (−0.73, 1.51)	0.071
	Adjusted model	1	−1.56 (−3.71, 0.58)	0.922	−0.11 (−2.24, 2.02)	0.153
WC (cm)	Crude model	1	−2 (−4.84, 0.84)	0.168	−3.35 (−6.32, −0.58)	0.019
	Adjusted model	1	−3.54 (−6.74, −0.34)	0.030	−2.09 (−5.25, 1.08)	0.197
HC (cm)	Crude model	1	−2.79 (−6.51, 0.91)	0.139	−4.25 (−7.99, −0.51)	0.026
	Adjusted model	1	−2.81 (−7.03, 1.41)	0.191	−4.37 (−8.51, 0.24)	0.490
WHR	Crude model	1	−2.85 (−7.38, 1.69)	0.535	−1.43 (−5.95, 3.09)	0.219
	Adjusted model	1	−2.53 (−7.28, 2.22)	0.651	−1.09 (−5.83, 3.64)	0.296
Sarcopenia	Crude model	1	0.46 (0.08, 0.83)	0.016	0.98 (−1.38, 0.59)	0.081
	Adjusted model	1	−1.84 (−4, 0.32)	0.731	−0.38 (−2.52, 1.77)	0.095
Absolute muscle strength (Kg)	Crude model	1	−0.91 (−1.38, −0.44)	0.025	−0.52 (−0.06, 0.98)	0.001
	Adjusted model	1	−1.88 (−4.06, 0.29)	0.696	−0.43 (−2.57, 1.73)	0.090
Relative muscle strength (m^2^)	Crude model	1	−0.62 (−1.59, 0.35)	0.125	0.76 (−0.21, 1.74)	0.208
	Adjusted model	1	−1.66 (−4.43, 1.1)	0.886	−0.21 (−2.95, 2.55)	0.238
SBP (mmHg)	Crude model	1	−0.17 (−1.88, 1.53)	0.151	1.26 (−0.46, 2.97)	0.843
	Adjusted model	1	−1.15 (−3.51, 1.21)	0.805	0.29 (−2.06, 2.65)	0.339
DBP (mmHg)	Crude model	1	−0.64 (−2.56, 1.28)	0.423	0.79 (−1.14, 2.71)	0.514
	Adjusted model	1	−1.5 (−3.99, 0.99)	0.963	−0.06 (−2.54, 2.42)	0.238

Note

The *p* values <0.05 was considered as significant with Logistic regression model by adjusting the age, sex and BMI, physical activity, and total calorie intake. The first tertile (T1) is considered as reference.

Abbreviations: DBP, diastolic blood pressure; DTAC, dietary total antioxidant capacity; FBS, fasting blood sugar; HC, Hip circumference; HDL, high density lipoprotein; LDL, low density lipoprotein; SBP, systolic blood pressure; TG, triglycerides; TC, total cholesterol; WC, waist circumference; WHR, waist to hip ratio.

Waist circumference values in the second tertile compared to the first one in the adjusted model was 3.54 lower (*p *= 0.030). Besides, the values of waist and hip circumference in the third tertile of the DTAC were significantly 3.35 and 4.25 times lower than the first tertile (*p *= 0.019, *p *= 0.026). In both models, the values of waist to hip ratio in the second and third tertiles were less than the first one, but the ratios were not significant. The crude model of sarcopenia in the second tertile was significantly smaller in comparison to the reference (*p *= 0.016), however, in the third tertile, the ratio was not significant. As well, the absolute muscle strength in the crude model of second and third tertiles was significantly weaker than the reference (*p *= 0.025, *p *= 0.001). The relative muscle strength, SBP, and DBP ratios were not significant in both models of second and third tertiles.

## 4 DISCUSSION

With the growth of the elderly population, the prevalence of type 2 diabetes as an age‐related disease also increases. In recent years several studies have demonstrated the bidirectional link between diabetes and sarcopenia. In this regard, there are various common mechanisms that link them to each other such as insulin resistance, oxidative stress, mitochondrial dysfunction, peripheral neuropathy and inflammatory cytokines (Khamseh et al., 2011). Although several studies have been performed to find the exact link between diabetes and sarcopenia, the role of nutritional factors has not yet been fully understood. This study was performed to determine the relationship between DTAC with sarcopenia and blood biomarkers in patients with type 2 diabetes. Our findings showed that the prevalence of sarcopenia between all participants was 57.1%, and the absolute muscle strength weakness was 36.5%. Also, a significant revers correlation was observed between DTAC and fasting blood sugar. In adjusted model, there was a non‐significant reverse relationship between the DTAC and values of profile lipids. Moreover, a reverse significant correlation was observed between DTAC and the values of waist circumference, hip circumference, and sarcopenia.

Unfortunately, the prevalence of sarcopenia among the participants of the present study was considerable. Similarly, in the Beretta et al. study, the prevalence of sarcopenia was 46.3% (Beretta et al., 2020) but it was 16.2% in Trierweiler et al. study (Trierweiler et al., 2018), and 13.6% in Pang et al. study, though it was 32.2% in people over 60 years old (Pang et al., 2021). Chronic hyperglycemia has an important role in accelerating the glycation products and vascular complications in various organs like SMM, by creating oxidative stress especially in diabetics (Beretta et al., 2020). Decreased SMM and strength in diabetics may depend on the duration of diabetes, the use of certain medications such as insulin sensitizers and dipeptidyl peptidase inhibitors (Galarregui et al., 2018; Goyal & Jialal, 2019).

In the current study, a significant inverse correlation was observed between DTAC and values of fasting blood sugar, but the relationship with serum lipids was not significant. Findings of different investigations have shown that people with diabetes have a lower DTAC compared to the control group (Hermsdorff et al., 2011; Psaltopoulou et al., 2011). In a study by Scott et al., a higher DTAC was significantly associated with a reduced risk of type 2 diabetes, and there was also a significant inverse relationship between DTAC and insulin resistance (Scott et al., 2017). Likewise, in another study that examined the effect of antioxidant supplements on the overall antioxidant status of children with type 1 diabetes, there was a statistically significant relationship between DTAC with fasting blood sugar and HbA1c (Parthasarathy et al., 2019). Also, an inverse relationship was observed between the consumption of antioxidant food sources and blood TG levels in Korean adults (Kim et al., 2019). It seems that oxidative factors have an important role in the progression of type 2 diabetes by increasing the production of free radicals and hypersensitivity of pancreatic cells to reactive oxygen species, mitochondrial dysfunction, apoptosis of pancreatic cells, resulting in impaired insulin secretion and regulation of blood glucose concentration (van der Schaft et al., 2019). Low DTAC, as an effective nutritional factor, can be a potential predictor of glucose intolerance in type 2 diabetes. In this way, the effects of food antioxidants on blood biomarkers can be through changes in fat and carbohydrate metabolism as well as increased insulin sensitivity (Mohammadi et al., 2021; Petelin et al., 2017; van der Schaft et al., 2019). The different results obtained in the present study may be due to its cross‐sectional design and relatively small sample size.

Furthermore, our findings showed that there is an inverse relationship between the DTAC tertile and the values of waist and hip circumference in the crude model. In this context, higher DTAC seems to be inversely related to anthropometric indices. Some studies have shown that the presence of inflammatory conditions caused by oxidative stress is due to insufficient consumption of dietary antioxidant sources (Hermsdorff et al., 2011; Mohammadi et al., 2021; Mozaffari et al., 2018). Some studies have also demonstrated that exposure to reactive oxygen species (ROS) can stimulate the differentiation and proliferation of fat cells, and the excess fat leads to the production of more ROS, this defective cycle amplifies obesity and may increase the likelihood of diabetes (Lee et al., 2009). In this regard, Gaman et al., have shown that the oxidative stress in type 2 diabetes is significantly associated with higher values of BMI, fat mass, and obesity in their study (Gaman et al., 2019). Besides, Ramezani et al. have indicated that DTAC was inversely associated with BMI (Ramazani et al., 2019). On the other hand, Aune et al. showed that the total antioxidant capacity of serum was significantly more in overweight people (Aune et al., 2011) that these different results may be attributed to the differences in the design of these studies.

The relationship between DTAC and sarcopenia was significant just in the second tertile in comparison to the reference in the crude model and this relationship was not significant about muscle strength in all models. There are numerous studies that showed an inverse relationship between DTAC and sarcopenia. Kucukdiler et al. have reported that oxidative stress is significantly higher in sarcopenic people (Küçükdiler et al., 2019). As well, in the Bernabeu‐Wittel study, 21.8% of sarcopenic subjects had higher oxidative stress markers (Bernabeu‐Wittel et al., 2020). Some studies suggested that using antioxidant supplements in elderly people can significantly reduce the destructive effects of ROS on muscle mass and function (Damiano et al., 2019). It seems that the role of oxidative factors in the development and progression of sarcopenia increases by enhancing apoptotic signaling and protein degradation (Asmat et al., 2016; Mancini et al., 2018). Food sources of antioxidants, such as fat‐soluble vitamin E, can exert their effects by interfering with the lipid peroxidation chain reactions in cell membranes. Other water‐soluble antioxidants such as vitamin C can directly counteract free radicals and thus prevent oxidative damage to various tissues including skeletal muscles (Ryan et al., 2010).

In the present study, the association between DTAC and blood pressure was not observed. Conversely, in a study by Mazloom et al., which examined the effect of alpha‐lipoic acid as an antioxidant on SBP and DBP in people with type 2 diabetes, a significant reduction was observed in blood pressure in the alpha‐lipoic acid group compared to the placebo group (Mazloom & Ansar, 2009). Similarly, in the study of Kim et al., the dietary pattern containing legumes, nuts, vegetables, and fruits, which are rich in antioxidants, was associated with a lower prevalence of hypertension (Kim et al., 2019). The function of nitric oxide, a vasodilator mediator, can be impaired with oxidative stress and free radicals (Heitzer et al., 2001). The difference in our findings with other studies may be due to the age of participants and the duration of the disease in our study.

The first limitation of the present study is related to the nature of cross‐sectional studies in which it is not possible to find a cause‐and‐effect relationship. So it is required to conduct comprehensive clinical trials with a large sample size to infer causality. Another limitation of our study is the use of food frequency questionnaire as a retrospective diet assessment method that is based on subjects’ memory, which increases the likelihood of misreporting and may affect the findings.

One of the strengths of this study is the investigation of DTAC and sarcopenia as important factors whose role in type 2 diabetes is not well considered. The use of information from a cohort study on the Kurdish ethnicity and the study of local foods used by this population in the food frequency questionnaire are other strengths of the present study. Other limitations of this study are failure to measure HbA1c, lack of access to computing tomography scans to directly assess muscle quality, as well as the inability to modify all lifestyle components as confounding factors.

## 5 CONCLUSION

The results of this study indicated that DTAC may be associated with fasting blood sugar, anthropometric indices, and sarcopenia. Therefore, following a proper nutrition program to increase the intake of dietary antioxidants can be beneficial in type 2 diabetic patients. In this study, the prevalence of sarcopenia in diabetic patients was considerable and the screening of these patients for sarcopenia in medical centers is not currently implemented. Therefore, by modifying and implementing the relevant instructions in medical centers, it is possible to diagnose this disease and its related causes in a timely manner and prevent its progression in diabetic patients by using appropriate interventions. However, more longitudinal studies in patients with type 2 diabetes are needed to fully understand the role of DTAC and diets high in antioxidants in the development of sarcopenia.

## CONFLICT OF INTEREST

Authors have no conflict of interest.

## AUTHOR CONTRIBUTIONS

Nadya Baharirad: study subject inclusion, data acquisition and interpretation, data analysis and interpretation, manuscript preparation; Yahya Pasdar and Mostafa Nachvak: study design and conception, study subject inclusion, data acquisition and interpretation, critical revision of the manuscript for intellectual content, administrative support; Shayan Mostafai and Armin Naghipour: data analysis and interpretation, critical revision of the manuscript for intellectual content; Saeid Ghavamzadeh, Ali Soroush, and Amir Saber: study design and conception, data interpretation, critical revision of the manuscript for intellectual content; Hadi Abdollahzad: study design and conception, data interpretation, study supervision, administrative support, critical revision of the manuscript for intellectual content. All authors have approved the submitted manuscript.
